# Skin Malignancies Due to Anti-Cancer Therapies

**DOI:** 10.3390/cancers16111960

**Published:** 2024-05-22

**Authors:** Michela Starace, Luca Rapparini, Stephano Cedirian

**Affiliations:** 1 Dermatology Unit, IRCCS Azienda Ospedaliero-Universitaria di Bologna, 40138 Bologna, Italy; michela.starace2@unibo.it (M.S.); stephano.cedirian@studio.unibo.it (S.C.); 2 Department of Medical and Surgical Sciences, Alma Mater Studiorum University of Bologna, 40138 Bologna, Italy

**Keywords:** target therapy, radiotherapy, transplant, anti-cancer therapies, non-melanoma skin cancer, melanoma, review

## Abstract

**Simple Summary:**

Anti-cancer treatments often entail a spectrum of adverse effects, and among these, skin cancers have been under significant scrutiny. From traditional immunosuppressants such as methotrexate to chemotherapeutic agents such as fludarabine and hydroxyurea, as well as from new targeted therapies such as ibrutinib and JAK inhibitors to MAP kinase pathway inhibitors and sonic hedgehog pathway inhibitors, each treatment modality poses unique mechanisms of action and associated risks of skin cancer. In addition, the role of radiotherapy in inducing secondary skin cancers underscores the long-term consequences of therapeutic interventions. It is crucial to ascertain whether a specific therapy increases the risk of skin cancer so that appropriate screening measures can be implemented before and during treatment. Our comprehensive review not only investigates various medications and treatment modalities linked with heightened skin cancer risks, elucidating their mechanisms of action and relevant properties, but also proposes effective strategies for managing patients undergoing such therapies.

**Abstract:**

Skin cancers involve a significant concern in cancer therapy due to their association with various treatment modalities. This comprehensive review explores the increased risk of skin cancers linked to different anti-cancer treatments, including classic immunosuppressants such as methotrexate (MTX), chemotherapeutic agents such as fludarabine and hydroxyurea (HU), targeted therapies like ibrutinib and Janus Kinase inhibitors (JAKi), mitogen-activated protein kinase pathway (MAPKP) inhibitors, sonic hedgehog pathway (SHHP) inhibitors, and radiotherapy. MTX, a widely used immunosuppressant in different fields, is associated with basal cell carcinoma (BCC), cutaneous squamous cell carcinoma (cSCC), and cutaneous melanoma (CM), particularly at higher dosages. Fludarabine, HU, and other chemotherapeutic agents increase the risk of non-melanoma skin cancers (NMSCs), including cSCC and BCC. Targeted therapies like ibrutinib and JAKi have been linked to an elevated incidence of NMSCs and CM. MAPKP inhibitors, particularly BRAF inhibitors like vemurafenib, are associated with the development of cSCCs and second primary melanomas (SPMs). SHHP inhibitors like vismodegib have been linked to the emergence of cSCCs following treatment for BCC. Additionally, radiotherapy carries carcinogenic risks, especially for BCCs, with increased risks, especially with younger age at the moment of exposure. Understanding these risks and implementing appropriate screening is crucial for effectively managing patients undergoing anti-cancer therapies.

## 1. Introduction

Anti-cancer treatments can often induce a range of adverse effects, and among these, skin cancers have been the subject of considerable scrutiny in both traditional and novel therapeutic approaches [1,2,3,4,5]. It is crucial to determine whether a particular therapy heightens the risk of skin cancer to implement appropriate screening measures before and during treatment. This comprehensive review first aims to investigate various medications and treatment modalities associated with increased skin cancer risks, along with their mechanisms of action and relevant properties (Table 1). Second, it proposes proper strategy for managing patients undergoing such therapies.

## 2. Classic Immunosuppressants

### 2.1. Methotrexate

#### 2.1.1. Clinical Indications and Mechanism of Action of Methotrexate

MTX functions as an antimetabolite, impeding the folate-dependent processes necessary for synthesizing purines and thymidylate. MTX has dual roles in medical treatment; it is employed in anticancer therapeutic protocols targeting various conditions like osteosarcoma, breast cancer, and blood malignancies. Simultaneously, MTX functions as an anti-inflammatory agent, addressing ailments such as rheumatoid arthritis (RA), Crohn’s disease, and vasculitis, among other cutaneous diseases such as psoriasis or atopic dermatitis [59]. 

#### 2.1.2. Oncogenic Mechanism of Methotrexate

Notably, MTX not only exhibits immunosuppressive properties but is also associated with photosensitization, both of which are implicated in increased skin cancer risk [6,7,8,9]. 

Polesie et al. found that patients who had a cumulative MTX dose of 2.5 g or more had a higher risk of developing BCC, cSCC, and CM compared to those who did not use MTX. Notably, there was a dose–response relationship observed for BCC and cSCC, implying that the risk of these skin cancers increased with higher doses of MTX [8]. Analogously, Vanni et al., who randomized 2391 patients to receive either low-dose MTX or a placebo, also found an increased risk of skin cancer, particularly cSCC, in the MTX-treated group compared to the placebo group. However, it has been noted that skin cancer was not a primary endpoint of interest in their study, and the confidence intervals were wide, indicating some uncertainty in the estimates [11]. 

**Figure 1:**
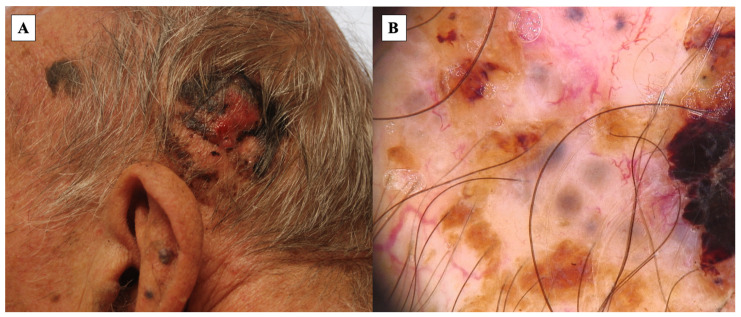
Clinical (**A**) and dermoscopic (**B**) images of a BCC located on the left temporoparietal area of a patient currently undergoing treatment with MTX for RA. The lesion has significantly progressed in size due to the patient’s non-compliance with follow-up appointments.

Lange et al. reported a 4.6-fold increase in the incidence of BCC or cSCC among patients with inflammatory arthritis using MTX compared to non-users. The risk escalates significantly with cumulative doses exceeding 8000 mg, demonstrating a dose–response relationship primarily concerning BCC (Figure 1) [10]. Although an increased risk of CM has been associated with MTX use, its clinical significance might be marginal. A cohort study revealed a threefold higher risk of CM among RA patients exposed to MTX compared to the general population. Nonetheless, without a control group of MTX-naive RA patients, discerning the attributable risk solely to MTX remains challenging [12]. 

Lastly, a recent meta-analysis of 17 studies identified a 15% increased risk of CM among participants exposed to low-dose MTX compared to alternative treatments. This study indicates that the percentage is not relevant to the risk–benefit assessment for patients with a low baseline risk of CM [13].

### 2.2. mTOR Inhibitors

#### 2.2.1. Clinical Indications and Mechanism of Action of mTOR Inhibitors

Immunosuppressive medications constitute a vital category of anti-cancer drugs widely utilized in the treatment of various solid tumors and blood-related conditions. Among these, inhibitors of the mTOR pathway, including sirolimus (rapamycin), everolimus, and temsirolimus, exert dual effects by suppressing the immune system and impeding cell proliferation. Upon antigen binding to a T cell receptor with CD28 co-stimulation or IL-2 receptor binding, the activated mTOR pathway triggers heightened protein synthesis for cellular division. Disrupting the formation of the mTOR complex, mTOR inhibitors impede T cells from progressing into the S phase of cell division, inducing a state of anergy in native T cells [14]. 

These medications are indispensable for preventing organ rejection post-transplantation, treating cancers such as renal cell carcinoma and transplant-associated Kaposi sarcoma, and managing other disorders characterized by excessive cell growth, like tuberous sclerosis. 

#### 2.2.2. Oncogenic Mechanism of mTOR Inhibitors

However, this mechanism remains uncertain whether blocking mTOR signaling also impedes the onset of skin cancer [1]. Data from the literature indicate that everolimus does not affect cell survival post-UV exposure, the cellular capacity for nucleotide excision repair (NER), or the elimination of cyclobutane pyrimidine dimer (CPD) and pyrimidine-6,4-pyrimidone photoproduct (6-4PP) lesions from cellular DNA. Consequently, mTOR inhibitors do not hinder NER, which is the principal mechanism for repairing UV-induced DNA damage [60]. On the other hand, rapamycin might impede transcription-coupled NER (TCR) to a certain degree, as was recently revealed in a yeast model system [15]. Nonetheless, as demonstrated in Cockayne syndrome, the lack of TCR does not result in increased susceptibility to photo-carcinogenesis [16]. In addition, Chen et al. demonstrated in breast cancer cells that rapamycin inhibits the two main pathways for repairing double-strand breaks [61]. This results in increased genomic instability within cells when exposed to endogenous factors (e.g., during S-phase) or exogenous factors (e.g., ionizing radiation) that induce strand breaks and, ultimately, may lead to an increased risk of skin malignancy [17].

## 3. Chemotherapeutic Agents 

### 3.1. Fludarabine

#### 3.1.1. Clinical Indications and Mechanism of Action of Fludarabine

Fludarabine, a purine analog, is an antimetabolite, antineoplastic agent used in treating various hematological malignancies, particularly B-cell chronic lymphocytic leukemia/small lymphocytic lymphoma (CLL/SLL). It inhibits DNA synthesis by targeting DNA polymerase and ribonucleotide reductase. Additionally, it inhibits DNA primase and DNA ligase I [62]. 

#### 3.1.2. Oncogenic Mechanism of Fludarabine

**Figure 2:**
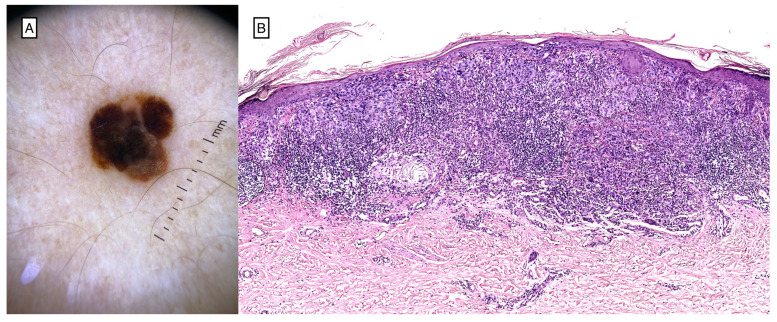
Dermoscopic image of a 1.1 mm CM situated on the chest of a patient currently undergoing therapy with Fludarabine for CLL/SLL (**A**). Histological picture of superficial spreading melanoma, vertical growth phase, infiltration thickness according to Breslow 1.1 mm, 2 mitoses/mm^2^ (H&E, 6×) (**B**).

In a population-based study, patients with CLL/SLL treated with fludarabine-containing chemotherapy were found to have approximately twice the risk of developing CM compared to survivors of non-CLL/SLL [21] (Figure 2). The exact mechanism by which fludarabine induces CM is unclear, but it may involve an inherent predisposition to malignancy combined with its immunosuppressive and DNA-damaging effects [18,19,20]. CMs occurring after CLL/SLL tend to be more advanced and aggressive (1 mm thick or greater), likely due to prolonged immunosuppression. Unlike CLL/SLL, there is limited evidence of increased CM risk after other non-Hodgkin lymphoma (NHL) subtypes or specific chemotherapy regimens, such as cyclophosphamide [21].

### 3.2. Hydroxyurea (HU)

#### 3.2.1. Clinical Indications and Mechanism of Action of Hydroxyurea

HU, also known as hydroxycarbamide, is a cytotoxic antimetabolite extensively used for managing various medical conditions. HU is utilized in treating sickle cell anemia, myeloproliferative disorders (such as polycythemia vera, essential thrombocythemia, and primary myelofibrosis), hyper-eosinophilic syndrome, and refractory psoriasis [22,63,64]. It works by inhibiting the ribonucleotide reductase enzyme, thus impeding the conversion of ribonucleotides into deoxyribonucleotides during the S phase of cellular division [22,27,63,64]. Consequently, it hampers cellular proliferation and serves as a cytoreductive treatment in Philadelphia chromosome-negative myeloproliferative neoplasms (Ph-MPN) cases [64]. 

#### 3.2.2. Oncogenic Mechanism of Hydroxyurea

**Figure 3:**
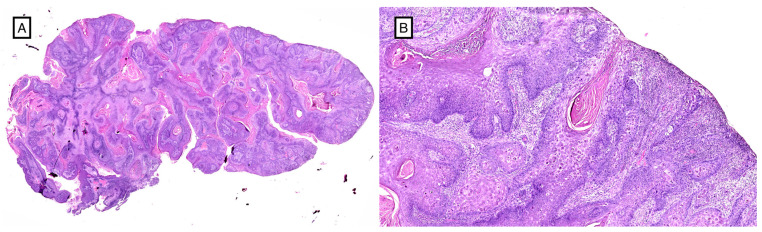
Histological picture of moderately differentiated cutaneous squamous cell carcinoma (H&E, 1×) (**A**). Squamous eddies, aggregations of atypical keratinocytes with large, hyperchromatic, pleomorphic nuclei and eosinophilic cytoplasm (H&E, 4×) (**B**).

Despite its generally favorable tolerability, the widespread usage of HU has brought to light adverse effects linked to tissues characterized by high cellular turnover, such as the skin. Several cutaneous adverse events have been documented in individuals receiving HU treatment [22,27]. Among these, NMSCs such as cSCC (Figure 3), BCC, and Merkel cell carcinoma (MCC) prompt the cessation of treatment due to unacceptable high levels, necessitating adjustments in treatment strategies. An elevated risk of actinic keratosis (AK), a pre-cancerous lesion, has also been linked with this treatment regimen; specifically, AK has been reported in 15 patients, often preceding the appearance of NMSC [22,27]. The median time to NMSC after initiating HU was 75 months (range 1–204) at a median HU daily dose of 1.25 g (range 0.5–2) [27]. According to available research findings, these cancers manifested in patients undergoing prolonged drug therapy [25,65]. The pathogenesis behind the augmented risk of skin malignancies is associated with UV exposure; specifically, HU inhibits DNA synthesis and repair, while UV radiation induces mutations in the p53 gene within keratinocytes, impairing its tumor-suppressing function. UV exposure activates mutated keratinocytes, facilitating their spread and increasing the risk of skin tumors [22,23,24]; indeed, these cancers primarily manifest on the scalp, ears, neck, hands, and feet [22,26,27,66], indicating a potential contributory role of excessive sunlight exposure in carcinogenesis. 

Gavini et al. incorporated a total of 140 reported cases from various reviews [22]. Patients diagnosed with Ph-MPN who are undergoing treatment with HU and subsequently develop skin cancers typically belong to the elderly demographic [67]. The median age recorded among patients is no less than 61 years [22]. No definitive correlation has been established between the occurrence of these cancers and gender. Both men and women exhibit equal vulnerability, although a study indicated a slight male predominance [22]. 

Among six observational studies, two concluded that HU does not induce NMSC [66,68], although the median HU dosage was not disclosed in either study; however, their collective evidence underscores a potential risk of NMSC among elderly Ph-MPN patients exposed to high doses of HU over extended periods, particularly in sun-exposed areas [22]. 

#### 3.2.3. Management of Patients in Therapy with Hydroxyurea

Patients on chronic HU medication should minimize outdoor activities, use photoprotective barriers, and consider chemopreventive agents such as oral retinoids to reduce the risk of skin tumors [25,26]. Surgical excision of the suspected lesion and HU discontinuation were the most frequent types of intervention [27]. Most lesions either improved or remained stable following discontinuation of the drug [25,28].

## 4. Targeted Therapies

### 4.1. Ibrutinib

#### 4.1.1. Clinical Indications and Mechanism of Action of Ibrutinib

Ibrutinib functions as a strong and permanent blocker of Bruton’s tyrosine kinase (BTK), a crucial element within both the B-cell receptor (BCR) and cytokine receptor pathways. Sustained activation of BCR signaling is critical for the survival of cancerous B-cells; inhibiting BTK leads to reduced proliferation and survival of malignant B-cells. Ibrutinib has demonstrated notable effectiveness and tolerability in managing various B-cell malignancies (e.g., CLL/SLL and Waldenström macroglobulinemia) and chronic graft-versus-host disease [9]. 

#### 4.1.2. Oncogenic Mechanism of Hydroxyurea

Despite exhibiting potential anti-skin cancer properties in laboratory studies, retrospective analysis from the Food and Drug Administration Adverse Event Reporting System (FAERS) revealed a substantially higher incidence of NMSC (6.5-fold) and CM (2.5-fold) reports associated with ibrutinib compared to other medications in the database. These associations persisted even when comparing ibrutinib with venetoclax, a BCL2 antagonist [9,29].

### 4.2. JAKi 

#### 4.2.1. Mechanism of Action of JAKi

Another category of drugs potentially linked to an increased incidence of NMSC is the JAKi family. Several proinflammatory signaling pathways converge on JAK family proteins, pivotal in cytokine signal transduction. They phosphorylate activated cytokine receptors, facilitating the recruitment and activation of transcription factors known as signal transducers and activators of transcription (STATs). Once phosphorylated, STATs dimerize and move to the nucleus, directly enhancing transcription. 

#### 4.2.2. Clinical Indications of JAKi

JAKi regulates the immune response by diminishing the impact of interleukin and interferon signaling. This broad mechanism accounts for their effectiveness in treating conditions such as inflammatory arthritis, IBD, psoriasis, alopecia areata, and vitiligo, as well as blood malignancies such as myeloproliferative disorders [3,69]. 

#### 4.2.3. Oncogenic Mechanism of JAKi

Concern arises regarding the risk of skin cancer, especially cSCC, in patients with myeloproliferative disorders treated with ruxolitinib. A retrospective cohort study revealed a significant increase in cSCC risk among ruxolitinib-exposed patients, particularly in those without a JAK2 mutation. Notably, a history of immunosuppression was strongly associated with NMSC risk, regardless of ruxolitinib intake [70]. The COMFORT-II trial examined the 5-year efficacy of ruxolitinib in patients with myelofibrosis. The study indicated that patients treated with ruxolitinib had a significantly higher incidence of NMSC compared to those receiving the best available therapy (17.1% vs. 2.7%). This increased risk persisted even after adjusting for patient exposure [31]. Additionally, an 80-week follow-up of a phase 3 clinical trial of ruxolitinib for polycythemia vera also found a higher occurrence of NMSC among ruxolitinib-treated patients compared to the control cohort. The rate of NMSC occurrence was higher in the ruxolitinib group, although the increase was less pronounced compared to the COMFORT-II trial [32]. 

A decade-long clinical study involving 564 participants indicated an elevated likelihood of cSCC occurrence following ruxolitinib therapy. Nonetheless, the precise mechanism through which ruxolitinib heightens cSCC risk remains unclear. It is hypothesized that JAKis such as ruxolitinib impede immune function, particularly the generation of IL-6, IL-23, and Th17 activity, which could promote tumor growth during cSCC progression [30]. Studies analyzing the efficacy of tofacitinib in cutaneous psoriasis and RA have shown a relatively low risk of NMSC associated with this JAKi. For example, two phase 3 trials for psoriasis demonstrated that only 2 out of 1486 patients treated with tofacitinib developed NMSC. Similarly, analysis across the tofacitinib RA clinical program showed an overall low incidence of NMSC, though higher doses may slightly elevate this risk [71]. 

On the other hand, a large, randomized trial focusing on RA patients aged 50 years and older showed a higher risk of both NMSC and other cancers with tofacitinib compared to TNF inhibitor therapy [33]. Although data from the literature have proven such an association between skin cancers and JAKis, in a comprehensive analysis of 17 controlled trials involving patients treated with JAKis such as tofacitinib, baricitinib, filgotinib, or upadacitinib for RA, cutaneous psoriasis, IBD, or ankylosing spondylitis, no increased risk of NMSC or other malignancies was observed compared to placebo or other active treatments [72]. The COMFORT-I trial evaluated the efficacy of ruxolitinib in myelofibrosis patients over a 5-year period and observed no increased risk for NMSC among patients treated with ruxolitinib compared to those on placebo, as well [73]. A study on the safety profile of upadacitinib in rheumatoid arthritis showed that the risk of developing NMSC was higher in patients receiving upadacitinib 30 mg/day compared to MTX, but was not statistically significant [34]. This discrepancy suggests that further investigation is needed to fully understand the relationship between JAKi and NMSC. As for CMs, they have been reported in patients treated with ruxolitinib [35], but no significant increase in CM incidence was observed compared to non-exposed patients with myeloproliferative disease [74,75]. 

An interesting finding emerges from some case reports linking JAKi therapy to the development of Kaposi sarcoma [36,37]. This phenomenon is probably related to the absolute decrease in CD4 and/or CD8 cell counts and the impairment of natural killer cell function induced by ruxolitinib, which could favor the reactivation of latent human herpes virus 8 or its acquisition [36].

#### 4.2.4. Management of Patients in Therapy with JAKi

Given these findings, routine skin cancer screening is recommended for patients with myeloproliferative disorders on ruxolitinib and for those with RA or psoriasis treated with JAKi. Additionally, in cases where cSCC develops while on JAKi therapy, reducing overall immunosuppression, including adjusting the JAKi dose, is advised [3,9]. 

### 4.3. MAPKP Inhibitors

The MAPKP, also known as the RAS/RAF/MAPK signaling pathway, plays a critical role in mediating communication between growth factors and receptors, which is essential for cell differentiation, survival, and proliferation. 

#### 4.3.1. Clinical Indications of MAPKP Inhibitors

Mutations within this pathway, particularly in the RAS oncogene and downstream BRAF, a serine/threonine kinase, enable cells to grow and proliferate independently of normal growth factor signals. In CM, the BRAF V600E mutation is particularly common, accounting for approximately 50% of CMs [48,49]. The accepted therapy for stage IV metastatic CM involves the use of BRAF inhibitors [76]. 

#### 4.3.2. Oncogenic Mechanism of MAPKP Inhibitors

However, a common occurrence with MAPKP inhibitors is the development of NMSCs. The existing literature proposes two main hypotheses to explain the underlying molecular mechanisms of such adverse events. The initial hypothesis suggests that BRAF inhibitors impede the MAPKP, particularly pERK, inducing cell cycle arrest and apoptosis and hindering tumor advancement in BRAF-mutated skin cancers. Conversely, in wild-type BRAF cells, activated ERK may stimulate abnormal cell proliferation, potentially leading to cSCC formation. Alternatively, the second theory posits that BRAF inhibitors induce keratinocyte hyperproliferation by paradoxically activating the MAPKP in cells with wild-type BRAF, triggering upstream-activating mutations in RAS or tyrosine kinase receptors. When exposed to a BRAF inhibitor, inactive BRAF forms heterodimers with wild-type CRAF, which leads to the transactivation of CRAF molecules by mutant RAS. As a result, this paradoxical increase in MAPK signaling leads to the phosphorylation and activation of ERK via CRAF signaling [2,3,38,39,40]. 

#### 4.3.3. Incidence of NMSC in Patients Treated with MAPKP Inhibitors

Vemurafenib, a selective BRAF inhibitor, gained approval from the US Food and Drug Administration (FDA) in 2011 for metastatic CM treatment. Clinical trials, including phase 3 studies, demonstrated that vemurafenib significantly improved survival rates and reduced disease progression compared to previous treatments such as dacarbazine. In addition, during phase 1 trials, vemurafenib treatment was associated with a notable 31% increased risk of cSCC development, particularly well-differentiated cSCCs and keratoacanthomas (KAs) [41]. Further studies, including phase 2 and 3 trials, confirmed the association between vemurafenib treatment and increased cSCC risk, albeit with a lower incidence [3]. 

McArthur et al. reported a 19% incidence of cSCC in extended follow-up of a phase 3 trial. In this study, cSCC developed around 10 weeks after starting vemurafenib therapy, with some cases appearing as early as 3 weeks into treatment. Notably, most patients with cSCC displayed clinical signs of chronically sun-damaged skin, despite only a minority having a history of cSCC before treatment [42,43]. RAS mutations, particularly HRAS mutations, have been identified in a significant proportion of vemurafenib-associated cSCCs and KAs. This suggests that vemurafenib may potentiate tumorigenesis in pre-existing subclinical lesions harboring upstream MAPKP mutations rather than inducing the development of new lesions [43]. 

Another paper on a meta-analysis encompassing 24 primary studies and involving 7442 patients revealed notable findings regarding the incidence of cSCC in individuals undergoing treatment with BRAF inhibitors. The study revealed that among cancer patients treated with BRAF inhibitors, the occurrences of all-grade and high-grade cSCC were 12.5% and 11.6%, respectively. Conversely, in those receiving dual BRAF/MEK inhibitors, the incidences were notably lower at 3.0% for all-grade cSCC and 2.8% for high-grade cSCC. Subgroup analysis and meta-regression revealed consistent cSCC incidence across various parameters, such as tumor type, study design, and specific drug used. Importantly, the use of single-agent BRAF inhibitors significantly elevated the risk of developing cSCC compared to dual BRAF/MEK inhibitors, with a substantial risk increase observed for both all-grade (RR 4.72, 95% CI: 2.42–9.20) and high-grade (RR 4.92, 95% CI: 2.64–9.16) cSCC in cancer patients [44]. Sorafenib, a multiple tyrosine kinase inhibitor that also inhibits the RAF serine/threonine kinases, used in treating solid tumors like hepatocellular and renal cell carcinomas, has also been associated with the development of cSCCs and KAs. Sorafenib has been implicated in inducing cSCCs shortly after starting treatment, especially in patients with chronic actinic damage. Considering the risk of cSCC development, it may be prudent to consider alternative tyrosine kinase inhibitors, such as sunitinib, for patients who develop multiple cSCCs while on sorafenib therapy [45,46,47]. 

#### 4.3.4. Incidence of SPMs in Patients Treated with MAPKP Inhibitors

In addition to cSCCs, SPMs have been reported in patients treated with BRAF inhibitors. These CMs are 10-fold less common than NMSC due to BRAFi and often arise in melanocytes with wild-type BRAF and may result from various signaling pathways beyond the RAF kinases [48,49,50]. 

#### 4.3.5. Management of Patients in Therapy with MAPKP Inhibitors

Despite the increased risk of cSCC, BRAF inhibitors remain highly efficacious in treating metastatic CMs harboring the V600E BRAF mutation. Treatment guidelines do not restrict their use, and cSCCs that develop during treatment tend to be low-grade, often managed with simple excision. Additionally, the combination of MEK inhibition with BRAF inhibitor therapy has been shown to reduce the risk of cSCC development [3].

Routine full-body skin examinations are recommended for patients receiving BRAF inhibitor therapy due to the increased risk of cSCC and SPM [3].

### 4.4. SSHPi

#### 4.4.1. Clinical Indications and Mechanism of Action of SSHPi

The FDA has recently authorized the use of vismodegib to hinder the SHHP in advanced BCC cases. In 2015, a second agent with a similar mechanism, sonidegib, received FDA approval for locally advanced BCC. About 90% of BCC cases exhibit mutations in the SHHP, resulting in constant smoothened activation and unregulated cell growth. The introduction of such inhibitors provided a crucial treatment option for inoperable or metastatic BCC; however, prolonged use may lead to drug resistance in some patients [2,3]. 

#### 4.4.2. Oncogenic Mechanism of SSHPi 

Reports have shown instances of KAs and cSCC emerging after vismodegib treatment for advanced or metastatic BCC [3,4,5]. For instance, Mohan et al. conducted a retrospective cohort study and reported a significant hazard ratio of 8.12 for cSCC occurrence among patients treated with vismodegib compared to standard BCC therapy [51]. The time frame for cSCC development varies, with clinical improvement or stability of the initial BCC sometimes observed prior to cSCC emergence; in most cases, signs of secondary cSCC appear within four months of vismodegib therapy initiation, even after initial complete BCC tumor response [5]. However, metastatic disease from cSCC has been documented only once; indeed, Zhao et al. presented a case of an advanced stage of secondary cSCC, notably with lymph node metastases. Possible factors contributing to this advanced stage include the patient’s immunosuppressed status, delayed presentation, and potential sampling error from the original BCC biopsy [5]. 

The exact cause of cSCC development following SHHP inhibition remains unclear. Evidence suggests that same-site cSCCs share many driver mutations with the original BCC, indicating a phenotypic switch rather than de novo development [3,5]. Kuonen et al. propose that the loss of primary cilia in vismodegib-resistant BCCs could link to cSCC development, as cilia paucity correlates with increased RAS/MAPK activity, a known oncogenic pathway in human cSCCs [52]. Further studies indicate that patients on vismodegib who develop cSCC at the original BCC site exhibit elevated ERK levels within tumor tissue, suggesting potential upregulation of the RAS/RAF/MAPK pathway during SHHP inhibition [5]. Other research on SHHP inhibition in medulloblastoma models also suggests activated RAS/RAF/MAPK pathways, implying that tumors under SHHP inhibition might activate alternative growth pathways to bypass the SHHP [3,51]. However, a recent meta-analysis confirmed that the occurrence of cSCC in patients taking vismodegib was not statistically significant [77]. A bias is represented by the fact that BCC and cSCC have common risk factors. One of the key findings is the importance of considering confounding variables such as the frequency of evaluation by a dermatologist. Patients receiving vismodegib, especially those participating in clinical trials, tend to undergo more frequent screenings for skin cancer. This heightened surveillance may lead to increased detection of cSCC, but it does not necessarily indicate a genuine increase in the risk of secondary cancer associated with vismodegib therapy [78]. 

Additional prospective studies are required to validate the correlation between vismodegib and cSCC, ascertain the frequency of cSCC occurrence, and identify potential pre-treatment variables, tumor characteristics, or therapeutic factors influencing cSCC emergence. Additionally, some research suggests that patients treated with vismodegib may have a lower propensity for developing cSCC in situ compared to control groups [51]. 

#### 4.4.3. Management of Patients in Therapy with SSHPi

Scheduled full-body skin examinations and careful surveillance are recommended for patients receiving vismodegib for advanced BCC treatment.

## 5. Radiotherapy 

For a considerable time, radiotherapy has been recognized to carry out carcinogenic risks. Essentially, radiation-induced secondary cancers are characterized as cancers that develop within or near areas exposed to radiation (field congruence), and they typically emerge after a considerable delay (greater than 10 years in certain studies, and even surpassing 15 years in others) [79]. Cases of skin cancers linked to radiation exposure have been documented among survivors of atomic bomb blasts, workers in uranium mines, medical professionals such as radiologists, and patients who underwent radiation therapy for both benign and malignant skin conditions [53,80,81,82,83]. Zelefsky et al. demonstrated elevated occurrences of skin cancer among individuals treated with external beam radiation therapy in contrast to the general population or those who underwent brachytherapy [84]. This phenomenon seems to stem from low-dose radiation exposure originating from internally scattered X-rays, leakage of X-rays from the machine, and/or neutron production, which is particularly noticeable following doses of 10 MV photons or higher [85]. 

### 5.1. Incidence of NMSC in Patients Treated with Radiotherapy

In a comprehensive study by Teepen et al. exploring the prevalence and risk factors associated with subsequent skin cancers among childhood cancer survivors (CCSs), researchers from the DCOG-LATER cohort shed light on critical insights. The cohort, comprising 5-year Dutch CCSs diagnosed between 1963 and 2001, revealed startling statistics. Among the 5843 CCSs studied, 259 individuals developed 1061 BCCs, indicating a standardized incidence ratio (SIR) of 29.8 (95% CI 26.3 to 33.6). In addition, the excess absolute risk per 10,000 person-years (EAR) stood at 24.6. Notably, the cumulative incidence of BCC 40 years after childhood cancer was 19.1% (95% CI 16.6 to 21.8%) for those subjected to radiotherapy, a stark contrast to the 0.6% expected based on general population rates. The study underscored a 30-fold increase in BCC risk among CCSs, with a notable association observed between BCC risk and the extent of exposed skin surface area. Specifically, individuals exposed to any radiotherapy targeting the skin compartment faced a hazard ratio (HR) of 14.32 (95% CI 10.10 to 20.29). Furthermore, the risk escalated with increasing percentages of in-field skin surface area, indicating a significant trend (P_trend_ among exposed = 0.002). The study findings from the DCOG-LATER cohort also illuminated a heightened risk of CM among CCSs compared to the general population. Chemotherapy analysis revealed a lower risk profile, with vinca alkaloids emerging as the sole agent associated with increased BCC risk (HR 1.54, 95% CI 1.04 to 2.27) [54]. 

### 5.2. Oncogenic Mechanism of Radiotherapy 

Most reports underscore the role of radiation in predominantly triggering BCC, with scant evidence supporting susceptibility to cSCC because of radiation exposure. Literature findings indicate that both single-fraction and escalating fractional doses of total body irradiation (TBI) elevate the risk of BCC, but not cSCC. These findings suggest that the basal layer of the epidermis is particularly responsive to radiation, and even with increasing cumulative doses, intervals between treatments may not facilitate complete repair [53,54]. In contrast, there is an absence of a gamma irradiation effect on cSCCs, which appear to be more prevalent following exposure to UV radiations or various chemical agents. Nonetheless, a recurring theme underscores the imperative of tissue and DNA repair mechanisms [53,86]. 

Another etiologic factor to consider when it comes to skin cancers due to radiation therapy is age at exposure. Previous studies have indicated that individuals exposed to radiation before the age of 10 face two to four times higher risks compared to their older counterparts; generally, the risk decreases by 10% for each year of age [55,56]. Observations from studies on second malignant neoplasms among CCSs (predominantly non-transplantation patients) show that a younger age at the time of radiation exposure heightened SMN risk. These collective findings suggest that children may possess greater inherent sensitivity to radiation [57,87]. 

### 5.3. Angiosarcomas Post-Radiotherapy 

Post-radiotherapy angiosarcoma is rare and develops in irradiation-exposed areas after several years. Iatrogenic angiosarcoma occurring in the context of breast cancer has been widely documented, and lymphoedema has been attributed as the main etiological factor. The incidence of radiation-associated angiosarcoma (RAAS) has increased with the spread of breast-conserving surgery. Although the characteristics of RAAS differ from primary angiosarcoma, the risk does not outweigh the benefits of adjuvant radiotherapy [58].

## 6. Conclusions

In conclusion, this comprehensive review underscores the intricate relationship between various anti-cancer treatments and the heightened risk of skin cancers. From classic immunosuppressants such as MTX to chemotherapeutic agents such as fludarabine and HU, as well as from targeted therapies such as ibrutinib and JAKis to MAPKP inhibitors and SHHP inhibitors, each treatment modality presents unique mechanisms and associated risks of skin cancer development. In addition, the role of radiotherapy in inducing secondary skin cancers, especially among CCSs, highlights the long-term implications of therapeutic interventions. Despite the advancements in cancer treatment, understanding and mitigating the risks of skin cancer associated with these treatments remain unclear. Therefore, clinicians should prioritize implementing appropriate screening measures and adopting proactive management strategies to ensure the holistic well-being of patients undergoing anti-cancer therapies. Additionally, ongoing research efforts are needed to further elucidate the underlying mechanisms and optimize treatment protocols to minimize the risk of skin cancers while maximizing therapeutic benefits. Finally, by fostering a comprehensive understanding of the intricate interplay between cancer treatments and skin cancer risks, healthcare practitioners can strive to provide optimal care and improve patient outcomes in oncology practice.

## Figures and Tables

**Table 1:** Summary of anticancer therapies, their mechanism, and associated skin malignancies.

Drugs	Oncogenic Mechanism	Associated Skin Malignancies
MTX	Immunosuppressive and photosensitizing action [6,7,8,9].	BCC [8,10] cSCC [8,10,11] CM [8,12,13]
mTOR inhibitors	Suppression of the immune system [14]. Mutagenesis in normal tissues [15]. Unsure whether to prevent the onset of skin cancer [1].	BCC [16,17] cSCC [1,16,17] CM [1,16,17] Mucosae melanoma [16]
Fludarabine	Inherent predisposition to malignancy, immunosuppressive and DNA-damaging effects [18,19,20].	CM [21]
HU	Inhibition of DNA synthesis and repair in cutaneous cells, inducing mutations [22,23,24].	BCC [25,26] cSCC [22,25,26,27,28] CM [22,27] MCC [22,25,27] AK [22,25,26,27,28] Bowen’s disease [25,28] Keratoacanthoma [25]
Ibrutinib	Increased photosensitivity [29].	NMSC [9,29] MC [9,29]
JAKi	Inhibition of immune function leads to the promotion of tumor growth [30].	BCC [31,32,33,34,35] cSCC [30,31,32,33,34,35] CM [35] Kaposi sarcoma [36,37]
MAPKPi	Proliferation in wild-type BRAF cells [2,3,38,39,40].	cSCC [2,3,38,39,40,41,42,43,44,45,46,47] Keratoacanthoma [41,43,45,46] SPM [48,49,50]
SSHPi	Inhibition of the hedgehog pathway activates the RAS/MAPK pathway, thus avoiding dependence on the hedgehog pathway for tumor growth [51,52].	cSCC [3,4,5,51] Keratoacanthoma [3,4,5]
Radiotherapy	Increased sensitivity of the basal layer of the epidermis to radiation [53,54].	BCC [53,54,55,56,57] Angiosarcoma [58]

AK: actinic keratosis; BCC: basal cell carcinoma; CM: cutaneous melanoma; cSCC: cutaneous squamous cell carcinoma; HU: Hydroxyurea; JAKi: Janus Kinase inhibitors; MAPKPi: mitogen-activated protein kinase pathway inhibitors MCC: Merkel cell carcinoma; MTX: methotrexate; NMSC: non-melanoma skin cancers; SPM: second primary melanoma; SSHPi: sonic hedgehog pathway inhibitors.
